# Clinical features and influencing factors analysis of T-cell large granular lymphocytic leukemia complicated with pure red cell aplasia

**DOI:** 10.3389/fimmu.2025.1695742

**Published:** 2026-01-21

**Authors:** Yiming Feng, Yufeng Du, Ying Wang, Yingying Ding, Jinsong Yan

**Affiliations:** 1 Department of Hematology, The Second Affiliated Hospital, Dalian Medical University, Dalian, China; 2 Department of Hematology, Liaoning Medical Center Hematopoietic Stem Cell Transplantation, the Second Hospital of Dalian Medical University, Dalian, China; 3 Department of Pediatric, Pediatric Oncology and Hematology Center, the Second Hospital of Dalian Medical University, Dalian, China; 4 Liaoning Key Laboratory of Hematopoietic Stem Cell Transplantation and Translational Medicine, Blood Stem Cell Transplantation Institute, the Second Hospital of Dalian Medical University, Dalian, China; 5 Dalian Key Laboratory of Hematology, Diamond Bay Institute of Hematology, the Second Hospital of Dalian Medical University, Dalian, China

**Keywords:** analysis of influencing factors, clinical features, complications, large granular T lymphocyte leukemia, pure red cell aplasia

## Abstract

**Objective:**

This study systematically characterized the key clinical features and influencing factors of patients with T-LGLL-PRCA, aiming to provide evidence to improve clinical diagnostic and therapeutic strategies.

**Methods:**

Clinical characteristics were retrospectively compared between patients with T-LGLL-PRCA (n=15) and those without PRCA (T-LGLL–Non-PRCA, n=25). Risk factors for the development of T-LGLL-PRCA were evaluated using univariate and multivariate logistic regression analyses.

**Results:**

This study retrospectively included a total of 40 patients with T-LGLL, of whom 15 were classified into the T-LGLL–PRCA group, accounting for one-third of the entire cohort. The median age of patients in the T-LGLL–PRCA group was 68 years, with a relatively high proportion aged ≥65 years, and the majority were female. The most common symptoms included fatigue, dizziness, and palpitations. The major comorbidity was thrombocytopenia. Positivity for EBV and ANA was frequently observed. Mutations in TET2 exon 11 and exon 3 were the most frequently detected genetic variants. At the molecular level, clonal rearrangements of the TCRβ and TCRγ genes were most commonly observed. Moreover, a substantial proportion of patients displayed a TCR γδ immunophenotype. Significant differences between groups were observed in circulating T-LGL count, Hemoglobin (HB), Hematocrit (HCT), Reticulocyte Count (RNC), Erythropoietin (EPO) levels >750 mIU/mL, and Immature Reticulocyte Fraction (IRF) (*P* < 0.05). Univariate logistic regression suggested that HB (*OR*:0.926; *P* = 0.003), HCT (*OR*:0.837;*P* = 0.006), EPO levels>750 mIU/mL (*OR*:7.071;*P* = 0.008), RNC (*OR*:0.682;*P* = 0.027), and IRF (*OR*:0.857;*P* = 0.007) were associated with T-LGLL-PRCA(*P* < 0.05). Multivariate analysis identified RNC (*OR*:0.590;*P* = 0.048) as the sole independent influencing factor (*P* < 0.05).

**Conclusion:**

RNC is an independent influencing factor for the development of T-LGLL-PRCA.

## 1 Introduction

T-cell large granular lymphocytic leukemia (T-LGLL) is a rare proliferative disorder of mature T lymphocytes, often associated with autoimmune disease and bone marrow failure (1, 2). Current studies suggest that T-LGLL represents a clonal lymphocyte proliferation process in which transient clonal expansions of T cells, triggered by infections (viruses), autoimmunity, and malignancies, overlap with reactive expansions (3, 4). However, the pathogenesis of T-LGLL is not yet fully understood. Current research indicates that it results from a combination of genetic susceptibility, abnormal immune regulation, and bone marrow microenvironment abnormalities, with the core issue being that clonal T cells escape normal regulation, leading to abnormal activation, proliferation, and resistance to apoptosis (5, 6). As an indolent disease, T-LGLL typically follows a slow clinical course, with common manifestations including autoimmune disorders and symptomatic cytopenias (7). More than half of affected individuals eventually develop hematologic and immunologic abnormalities requiring treatment, particularly moderate to severe cytopenias, which substantially impair prognosis and survival (5, 6). Unfortunately, no standardized first-line therapeutic strategy or guideline is currently available, and most patients are managed with immunosuppressive agents. However, these approaches offer limited success in eradicating malignant clones or sustaining long-term remission and fail to adequately address the poor prognosis associated with severe cytopenias.

Secondary pure red cell aplasia (PRCA) is one of the most common cytopenic complications in T-LGLL, with reported incidence rates being higher in Asian populations than in Western cohorts (8, 9). PRCA is a heterogeneous syndrome characterized by normocytic, normochromic anemia, markedly reduced reticulocyte counts, and a selective, severe reduction or absence of erythroid precursors in the bone marrow (10, 11). T-LGLL–associated PRCA (T-LGLL–PRCA) is a rare bone marrow failure disorder mediated by clonal T-LGLL cells. Patients frequently develop refractory anemia and transfusion dependence due to profound erythroid suppression and associated immune dysregulation, leading to markedly impaired quality of life and increased mortality risk (12, 13). Despite its clinical importance, T-LGLL–PRCA has not received sufficient attention. Owing to its rarity and unique pathophysiology, current research on T-LGLL–PRCA remains limited. Most published studies consist of isolated case reports or secondary descriptions within broader T-LGLL investigations, lacking robust cohort-based evidence (14). Furthermore, the factors contributing to the development of T-LGLL–PRCA—including clinical features, laboratory parameters, and molecular biomarkers—have not been clearly defined. This gap in knowledge limits early identification of high-risk patients and hinders the development of optimized therapeutic strategies.

In this study, patients with T-LGLL–PRCA were included as the primary research population, and a retrospective cohort design was used to analyze and compare demographic and clinical data while identifying potential risk factors. This framework provides a reliable basis for the early identification of high-risk T-LGLL–PRCA patients and for the development of individualized therapeutic strategies, ultimately contributing to improved quality of life and long-term outcomes.

## 2 Materials and methods

### 2.1 Study population

This single-center retrospective study included 40 patients diagnosed with T-LGLL between July 1, 2015, and June 30, 2025. Diagnosis was established according to the WHO Classification of Tumors of Hematopoietic and Lymphoid Tissues (5th Edition, 2022) and the Chinese Expert Consensus on Acquired PRCA (2020) (15, 16). Although no universally accepted diagnostic standard exists for T-LGLL, current clinical practice emphasizes integrating morphology, immunophenotype, TCR clonality, and relevant laboratory findings, consistent with the framework proposed by Marchand et al (4).

### 2.2 Study design

Data collected at first presentation included demographics, clinical symptoms, comorbidities, spleen size, hematologic parameters, EPO levels, reticulocyte indices (RNC, IRF), immunologic markers (EBV, CMV, HSV, ANA, RF, ENA, ANCA), flow cytometric phenotyping, TCR gene rearrangement patterns, TCR αβ/γδ subtype, and next-generation sequencing–based mutation results. Treatment strategies and responses were systematically recorded. Patients were categorized into the T-LGLL-PRCA group and T-LGLL-Non-PRCA group based on diagnostic criteria.

### 2.3 Statistical analysis

Statistical analyses were performed using SPSS 26.0. Continuous variables were assessed for normality using the Shapiro–Wilk test. Normally distributed data were expressed as mean ± SD and compared with the t-test; non-normal data were presented as median (interquartile range) and compared using the Mann–Whitney U test. Categorical variables were compared using Fisher’s exact test. Logistic regression was performed to identify factors associated with PRCA. Variables with *P* < 0.05 in univariate analysis were included in multivariate regression. *P* < 0.05 was considered statistically significant.

## 3 Results

### 3.1 Clinical characteristics of patients with T-LGLL-PRCA

#### 3.1.1 Baseline characteristics

A total of 40 patients with T-LGLL were included, of whom 15 were classified into the T-LGLL-PRCA group and 25 into the T-LGLL–Non-PRCA group. The overall median age was 66 years, and 57.5% of patients were aged ≥65 years. The T-LGLL-PRCA group exhibited a higher median age and a greater proportion of older adults than the T-LGLL–Non-PRCA group (68 years/73.3% vs. 61 years/48%). Females predominated in both the overall T-LGLL cohort and the T-LGLL-PRCA group (55% and 66.7%, respectively), whereas males were more frequent in the T-LGLL–Non-PRCA group (52%). A circulating T-LGL count ≥2.0 × 10^9^/L was observed in 22.5% of all patients and occurred more often in the T-LGLL–Non-PRCA group than in the T-LGLL-PRCA group (32% vs. 6.7%). Bone marrow T-LGL proportions ≥10% were observed in 52.5% of all patients, with a lower proportion in the T-LGLL-PRCA group than in the T-LGLL–Non-PRCA group (46.7% vs. 56%). Neutropenia (ANC <1.5 × 10^9^/L or <0.5 × 10^9^/L) was observed in 62.5% and 20% of patients, with no significant differences between groups. Thrombocytopenia occurred in 12.5% of patients, with comparable proportions between the two groups. Increased lymphocyte counts (ALC >4.0 × 10^9^/L) were identified in 20% of patients and were markedly more frequent in the T-LGLL–Non-PRCA group (32%) than in the T-LGLL-PRCA group (0%) (**Table 1**).

**Table 1:** Clinical characteristics of patients with T-LGLL-PRCA.

Items	Total T-LGLL(n=40)	PRCA(n=15)	Non-PRCA(n=25)
Age (years)			
Median (Min-Max)	66 (42-80)	68 (48-80)	61 (42-80)
≥65	23/40 (57.5%)	11/15 (73.3%)	12/25 (48%)
Sex (n, %)			
Male	18/40 (45%)	5/15 (33.3%)	13/25 (52%)
Female	22/40 (55%)	10/15 (66.7%)	12/25 (48%)
Circulating T-LGL (×10^9^/L)			
<2.0	31/40 (77.5%)	14/15 (93.3%)	17/25 (68%)
≥2.0	9/40 (22.5%)	1/15 (6.7%)	8/25 (32%)
BM T-LGL (%)			
<10	19/40 (47.5%)	8/15 (53.3%)	11/25 (44%)
≥10	21/40 (52.5%)	7/15 (46.7%)	14/25 (56%)
ANC (×10^9^/L)			
<1.5	25/40 (62.5%)	9/15 (60%)	16/25 (64%)
<0.5	8/40 (20%)	2/15 (13.3%)	6/25 (24%)
PLT (×10^9^/L) <100	5/40 (12.5%)	2/15 (13.3%)	3/25 (12%)
ALC (×10^9^/L) >4.0	8/40 (20%)	0	8/25 (32%)
Symptomatic (n, %)			
Fatigue	28/40 (70%)	14/15 (93.3%)	14/25 (56%)
Dizziness	13/40 (32.5%)	6/15 (40%)	7/25 (28%)
Chest Tightness	7/40 (17.5%)	2/15 (13.3%)	5/25 (20%)
Palpitation	6/40 (15%)	3/15 (20%)	3/25 (12%)
Fever	7/40 (17.5%)	1/15 (6.7%)	6/25 (24%)
Abdominal Distension	4/40 (10%)	1/15 (6.7%)	3/25 (12%)
Complications (n, %)			
Infectious Fever	7/40 (17.5%)	1/15 (6.7%)	6/25 (24%)
Thrombocytopenia	4/40 (10%)	3/15 (20%)	1/25 (4%)
Pancytopenia	3/40 (7.5%)	1/15 (6.7%)	2/25 (8%)
Pneumonia	3/40 (7.5%)	0	3/25 (12%)
Autoimmune Disease	1/40 (2.5%)	0	1/25 (4%)
Splenomegaly (n, %)	6/40 (15%)	1/15 (6.7%)	5/25 (20%)

#### 3.1.2 Clinical manifestations

The most common clinical manifestations in the overall T-LGLL cohort included fatigue (70%), dizziness (32.5%), chest tightness (17.5%), fever (17.5%), palpitations (15%), and abdominal distension (10%). Among patients with T-LGLL-PRCA, fatigue (93.3%), dizziness (40%), and palpitations (20%) occurred more frequently. In contrast, fever (24%), chest tightness (16%), and abdominal distension (12%) occurred more often in the T-LGLL–Non-PRCA group. The main comorbidities among all patients included infectious fever (17.5%), thrombocytopenia (10%), pancytopenia (7.5%), pneumonia (7.5%), and autoimmune diseases such as Sjögren’s syndrome (2.5%). Thrombocytopenia was more common in the T-LGLL-PRCA group (20%), whereas infectious fever, pancytopenia, pneumonia, and autoimmune diseases were more frequently observed in the T-LGLL–Non-PRCA group. Splenomegaly was observed in 15% of all patients and occurred more frequently in the T-LGLL–Non-PRCA group (20%) than in the T-LGLL-PRCA group (6.7%) (**Table 1**).

#### 3.1.3 Immune-related factors

PRCA, primarily involving viral infections and autoimmune antibodies. Among all patients, 18 had complete data for both viral infection screening and autoimmune antibody testing, including 8 in the T-LGLL-PRCA group and 10 in the T-LGLL–Non-PRCA group. The viral infections assessed in this study included Epstein–Barr virus (EBV), cytomegalovirus (CMV), and herpes simplex virus (HSV). Due to technical limitations, parvovirus B19 testing was not performed in this cohort. The results showed that, in the overall T-LGLL population, viral positivity rates ranked in descending order as EBV, CMV, and HSV (66.7%, 55.6%, and 38.9%, respectively). The positivity rates for all three viruses were consistently higher in the T-LGLL-PRCA group than in the T-LGLL–Non-PRCA group (87.5%/62.5%/50% vs. 50%/50%/30%), with the most pronounced difference observed for EBV (**Table 2**).

**Table 2:** Immune factors of patients with T-LGLL-PRCA.

Items	Total T-LGLL(n=18)	PRCA(n=8)	Non-PRCA(n=10)
Viral Infection (n, %)			
EBV (+)	12/18 (66.7%)	7/8 (87.5%)	5/10 (50%)
CMV (+)	10/18 (55.6%)	5/8 (62.5%)	5/10 (50%)
HSV (+)	7/18 (38.9%)	4/8 (50%)	3/10 (30%)
Autoimmune antibody (n, %)			
RF (+)	4/18 (22.2%)	1/8 (12.5%)	3/10 (30%)
ANA (+)	13/18 (72.2%)	7/8 (87.5%)	6/10 (60%)
ENA (+)	3/18 (16.7%)	1/8 (12.5%)	2/10 (20%)
ANCA (+)	2/18 (11.1%)	0	2/10 (20%)

For autoimmune antibodies, the assays performed included RF, ANA, ENA, and ANCA. In the overall T-LGLL group, ANA had the highest positivity rate (72.2%), followed by RF (22.2%), ENA (16.7%), and ANCA (11.1%). ANA positivity was markedly higher in the T-LGLL-PRCA group than in the T-LGLL–Non-PRCA group (87.5% vs. 60%). In contrast, RF, ENA, and ANCA positivity rates were higher in the T-LGLL–Non-PRCA group than in the T-LGLL-PRCA group (30% vs. 12.5%, 20% vs. 12.5%, and 20% vs. 0%, respectively) (**Table 2**).

### 3.2 Erythroid differentiation characteristics in T-LGLL-PRCA patients

T-LGLL-PRCA, a classical immune-mediated subtype of PRCA, is primarily driven by immune attacks mediated by aberrantly activated CD3^+^CD8^+^T cells or anti-EPO autoantibodies. These immune components collectively disrupt the differentiation of erythroid progenitors into early erythroblasts, resulting in selective suppression of erythropoiesis. Erythropoiesis is a continuous and dynamic process in which early progenitor cells mature into reticulocytes and ultimately into erythrocytes;different stages can be reflected by specific laboratory markers. Erythropoietin (EPO) reflects hypoxia-induced proliferative and differentiation-initiating signals for early erythroid progenitors (BFU-E/CFU-E) and therefore serves as an early regulatory marker of erythropoiesis. Reticulocyte count (RNC) and the immature reticulocyte fraction (IRF) indicate the production and maturation of mid-to-late-stage reticulocytes and provide insight into marrow reticulocyte output and stress erythropoiesis. Hemoglobin (HB) and hematocrit (HCT) reflect the quantity and functional output of terminally mature erythrocytes, representing the final stage of erythropoiesis. Together, these parameters constituted the erythroid assessment framework used in this study (17, 18).

The results showed that, in the overall T-LGLL cohort, the proportions of mild-tomoderate anemia (HB <120 g/L and <90 g/L), decreased HCT (HCT <35% and <20%), elevated EPO levels (>750 mIU/mL), reduced reticulocyte count (RNC <25 × 109/L), and decreased IRF (<10%) were 92.5%/72.5%, 90%/47.5%, 395 47.5%, 60%, and 40%, respectively. In the T-LGLL-PRCA group, these abnormalities occurred at higher frequencies—100%/100%, 100%/66.7%, 73.3%, 100%, and 66.7%, respectively—whereas the corresponding proportions in the T-LGLL–Non-PRCA group were 88%/56%, 84%/36%, 32%, 36%, and 24% (**Table 3**). Overall, all erythroid indicators showed a more pronounced decline in the T-LGLL-PRCA group, particularly HB <120 g/L, HB <90 g/L, HCT <35%, and RNC <25 × 10^9^/L.

**Table 3:** The erythroid differentiation indicators in patients with T-LGLL-PRCA.

Items	Total T-LGLL(n=40)	PRCA(n=15)	Non-PRCA(n=25)
HB (g/L)			
<120	37/40 (92.5%)	15/15 (100%)	22/25 (88%)
<90	29/40 (72.5%)	15/15 (100%)	14/25 (56%)
HCT (%)			
<35	36/40 (90%)	15/15 (100%)	21/25 (84%)
<20	19/40 (47.5%)	10/15 (66.7%)	9/25 (36%)
EPO (mIU/mL) >750	19/40 (47.5%)	11/15 (73.3%)	8/25 (32%)
RNC (×10^9^/L) <25	24/40 (60%)	15/15 (100%)	9/25 (36%)
IRF (%)<10	16/40 (40%)	10/15 (66.7%)	6/25 (24%)

### 3.3 Molecular biological characteristics of T-LGLL-PRCA patients

#### 3.3.1 Immunophenotype

T-LGLL cells, as clonally expanded mature effector–memory T cells, exhibit characteristic immunophenotypic patterns but also considerable heterogeneity. Typically, they display high expression of the pan–T-cell marker CD3 and predominantly exhibit a CD8^+^ phenotype, often accompanied by CD57^+^ expression (1, 4). However, atypical phenotypes, including CD4^+^ or double-negative CD4^-^CD8^-^ profiles, may also be observed (19, 20). Moreover, CD57 expression is not universal, and a minority of patients may show CD57 loss or aberrant expression of molecules such as CD2 or CD16 (21, 22).

In the overall T-LGLL cohort, the proportions of CD8^+^, CD4^+^, CD57^+^, CD16^+^, and TRBC1^+^ expression were 87.5%, 12.5%, 65%, 55%, and 25%, respectively, with CD8^+^ expression being the most prevalent. In the T-LGLL-PRCA subgroup, the corresponding frequencies were 86.7%, 13.3%, 60%, 46.7%, and 20%, respectively. Compared with the T-LGLL-PRCA subgroup, the T-LGLL–Non-PRCA group demonstrated higher proportions of CD8^+^, CD57^+^, CD16^+^, and TRBC1^+^ expression (88%, 68%, 60%, and 28%, respectively), whereas the proportion of CD4^+^ cells was slightly lower (12%) (**Table 4**).

**Table 4:** Immunophenotype analysis of patients with T-LGLL-PRCA.

Immunophenotype	Total T-LGLL(n=40)	PRCA(n=15)	Non-PRCA(n=25)
CD3 CD8 expression	35/40 (87.5%)	13/15 (86.7%)	22/25 (88%)
CD3 CD4 expression	5/40 (12.5%)	2/15 (13.3%)	3/25 (12%)
CD57 expression	26/40 (65%)	9/15 (60%)	17/25 (68%)
CD16 expression	22/40 (55%)	7/15 (46.7%)	15/25 (60%)
TRBC1 expression	10/40 (25%)	3/15 (20%)	7/25 (28%)

#### 3.3.2 Gene mutations

Somatic mutations in STAT3 and STAT5B have been identified in approximately 40% of patients with T-LGLL and are considered the most common genetic abnormalities;however, their association with T-LGLL-PRCA remains inconclusive (14, 23). This study also explored this issue, with particular interest in determining whether hematopoietic clones harboring specific gene mutations may contribute to marrow failure. Because genetic testing is not yet required for diagnosis and regional economic limitations existed, only 10 of the 40 patients underwent mutation profiling, including 6 in the T-LGLL-PRCA group. Hotspot mutation analysis for peripheral T-cell lymphoma–associated genes was performed using next-generation sequencing and bioinformatic pipelines, with bone marrow EDTA whole blood serving as the test specimen. Importantly, sequencing was performed on bone marrow mononuclear cells containing both myeloid and lymphoid components rather than on purified T-cell clones.

In the overall T-LGLL cohort, the most frequent mutation was TET2 exon 11 (40%), followed by TET2 exon 3 (30%), DNMT3A exon 18 (20%), TET2 exon 6 (10%), STAT3 exon 21 (10%), and JAK3 exon 17 (10%). In the T-LGLL-PRCA subgroup, TET2 exon 11 and exon 3 mutations predominated (66.7% and 50%, respectively), and two patients harbored both mutations concurrently. Additional mutations included DNMT3A exon 18 (33.3%), STAT3 exon 21 (16.7%), and JAK3 exon 17 (16.7%), whereas no TET2 exon 6 mutations were detected. In contrast, only TET2 exon 6 mutations (25%) were detected in the T-LGLL–Non-PRCA group, and no additional pathogenic mutations were identified. Overall, the T-LGLL-PRCA group exhibited a higher prevalence and broader spectrum of pathogenic mutations; however, given the small sample size, these findings should be interpreted as exploratory and supplementary (**Table 5**).

**Table 5:** Gene mutation analysis of patients with T-LGLL-PRCA.

Gene mutation	Total T-LGLL(n=10)	PRCA(n=6)	Non-PRCA(n=4)
TET2 (exon 11)	4/10 (40%)	4/6 (66.7%)	0
TET2 (exon 3)	3/10 (30%)	3/6 (50%)	0
TET2 (exon 6)	1/10 (10%)	0	1/4 (25%)
DNMT3A (exon18)	2/10 (20%)	2/6 (33.3%)	0
STAT3(Exon 21)	1/10 (10%)	1/6 (16.7%)	0
JAK3 (exon 17)	1/10 (10%)	1/6 (16.7%)	0
Negative	3/10 (30%)	0	3/4 (75%)

#### 3.3.3 TCR gene rearrangement and TCR subtype

Although the current diagnostic gold standard for T-LGLL is the demonstration of a clonal T-cell population by T-cell receptor (TCR) gene rearrangement analysis, it should be noted that a proportion of patients in our cohort were diagnosed in earlier years, when routine molecular assessment of TCR clonality was not universally available. In these cases, the diagnosis was established based on the then-accepted comprehensive criteria, including a persistent expansion of large granular lymphocytes, a characteristic cytotoxic T-cell immunophenotype, typical clinical manifestations, and exclusion of reactive or secondary causes. Importantly, these patients exhibited clinical courses and immunological features indistinguishable from those of cases with confirmed TCR clonality, supporting the reliability and homogeneity of the cohort. Therefore, although TCR rearrangement data were unavailable in a subset of historical cases, their inclusion is consistent with established diagnostic practice and is unlikely to introduce significant diagnostic bias (24, 25).

##### 3.3.3.1 TCR gene rearrangement

TCR gene rearrangements were assessed using a PCR-based GeneScan assay targeting the TCRG, TCRB, and TCRD genes. Bone marrow EDTA-anticoagulated whole blood was used as the sample source, from which total genomic DNA was extracted from mononuclear cells. Flow cytometry–based analysis of TCR Vβ repertoire was not performed in this study.

All patients in our cohort demonstrated a dominant clonal T-cell population, supporting the clonal nature of T-LGLL. Clonal rearrangements were detectable at either a single TCR gene locus or multiple loci, and positivity at multiple TCR loci reflects molecular detection of the same dominant clone across different TCR gene regions rather than true polyclonality.15 T-LGLL patients underwent TCR gene rearrangement testing, including 9 cases of T-LGLL-PRCA. In the overall T-LGLL group, TCR-β(+) and TCR-β/γ(+) had the highest proportion (both 26.7%), followed by TCR-β/γ/δ(+)(20%), TCR-γ(+)(13.3%), and TCR-δ(+)(13.3%); in the T-LGLL-PRCA group, TCR-β/γ(+) was highest (33.3%), followed by TCR-β(+)(22.2%) and TCR-γ(+)(22.2%); in the T-LGLL-Non PRCA group, TCR-β(+) and TCR-β/γ/δ(+) were highest (both 33.3%), followed by TCR-δ(+)(16.7%) and TCR-β/γ(16.7%) (**Table 6**). Overall, the proportions of TCR-β/γ (+) and TCR-γ(+) in the T-LGLL-PRCA group were higher than in the T-LGLL-Non PRCA group, while TCR-β(+), TCR-δ(+), and TCR-β/γ/δ(+) were the opposite. However, due to the small sample size, the above results can only be used as reference and supplementary information (**Table 6**).

**Table 6:** TCR Gene Rearrangement analysis of patients with T-LGLL-PRCA.

TCR gene rearrangement	Total T-LGLL(n=15)	PRCA(n=9)	Non-PRCA(n=6)
TCR-β (+)	4/15 (26.7%)	2/9 (22.2%)	2/6 (33.3%)
TCR-γ (+)	2/15 (13.3%)	2/9 (22.2%)	0
TCR-δ (+)	2/15 (13.3%)	1/9 (11.1%)	1/6 (16.7%)
TCR-β/γ (+)	4/15 (26.7%)	3/9 (33.3%)	1/6 (16.7%)
TCR-β/γ/δ (+)	3/15 (20%)	1/9 (11.1%)	2/6 (33.3%)

##### 3.3.3.2 TCR subtype

As a disease characterized by clonal T-cell receptor (TCR) gene rearrangement, CD3^+^ T-LGLL can be categorized into Tαβ and Tγδ subgroups based on the TCR rearrangement pattern. Tαβ-LGLL typically exhibits a CD8^+^/CD4^-^ phenotype (CD8^+^ T-LGLL), although CD4+/CD8- variants (CD4+ T-LGLL) have also been reported but are less common (4, 26).

In this study, 25 patients were evaluated for TCR-αβ and TCR-γδ subtype expression. The TCR-αβ subtype was the most frequent in both the overall T-LGLL cohort (60%) and the T-LGLL–Non-PRCA group (78.6%). Conversely, the TCR-γδ subtype predominated in the T-LGLL-PRCA group, with a frequency of 63.7% (**Table 7**).

**Table 7:** TCR Subtype analysis of patients with T-LGLL-PRCA.

TCR Subtype	Total T-LGLL(n=25)	PRCA(n=11)	Non-PRCA(n=14)
TCR-αβ (+)	15/25 (60%)	4/11 (36.4%)	11/14 (78.6%)
TCR-γδ (+)	10/25 (40%)	7/11 (63.6%)	3/14 (21.4%)

### 3.4 Treatment responses of T-LGLL-PRCA patients

T-LGLL is not a highly aggressive disease and typically follows an indolent clinical course. Approximately one-third of patients remain asymptomatic throughout the disease course, a condition referred to as T-cell colonopathy of unknown significance (TCUS) (27). Asymptomatic individuals are usually managed with watchful waiting until symptomatic cytopenias, splenomegaly, or other systemic manifestations emerge that warrant therapeutic intervention. For symptomatic patients, immunosuppressive therapy remains the mainstay of treatment, whereas targeted or salvage therapies may be considered in refractory or relapsed disease. Owing to the absence of randomized prospective trials, current treatment recommendations largely rely on phase II studies and pooled analyses of case series. First-line therapy generally consists of chronic, long-term immunosuppressive regimens, with methotrexate (MTX), cyclosporine A (CsA), and low-dose cyclophosphamide (CTX), with or without tapering glucocorticoids (GCs), being the most extensively studied agents (1, 4, 28).

In our center, treatment strategies included CsA monotherapy, CsA combined with steroids, CsA combined with CTX, and steroid monotherapy. Treatment response was evaluated based on hematologic improvement using predefined criteria for complete remission (CR), partial remission (PR), and no response (NR). Response assessments were performed after at least three months of therapy, and responses were required to persist for a minimum of four months to avoid misclassification due to transient fluctuations. CR was defined as normalization of all hematologic parameters, including ANC>1.5×10^9^/L, HB>110g/L without transfusion dependence, and PLT >150 × 10^9^/L. PR required significant hematologic improvement not meeting CR criteria, fulfilling any of the following for ≥3 months: >50% increase in ANC from baseline, >10 g/L increase in HB, or >50% reduction in transfusion requirements. NR was defined as failure to achieve CR or PR, lack of hematologic improvement, transient improvement followed by relapse within three months, or unchanged or increased transfusion dependence (4, 26).

Because patients with mild disease or those achieving remission often did not return for follow-up, complete post-treatment data were unavailable for many individuals. Among the 40 patients included in this study, complete clinical data at four months post-treatment were available for 21 patients—10 in the T-LGLL-PRCA subgroup and 11 in the T-LGLL–Non-PRCA subgroup. CsA combined with steroids is the most used treatment regimen among the three groups. The overall response rate (ORR) and complete remission rate (CRR) for the entire cohort were 57.1% and 19%, respectively. In the T-LGLL-PRCA subgroup, ORR and CRR were 60% and 20%, respectively, whereas the T-LGLL–Non-PRCA subgroup demonstrated an ORR of 54.5% and a CRR of 18.2%. However, owing to the relatively small sample size, the findings presented above should be considered exploratory. The influence of individual heterogeneity on treatment response cannot be fully ruled out, and these results are insufficient to provide direct or definitive guidance for the clinical treatment of T-LGLL-PRCA (**Table 8**).

**Table 8:** Treatment analysis of patients with T-LGLL-PRCA.

Treatment Response	Total T-LGLL(n=21)			PRCA(n=10)			Non-PRCA(n=11)		
	N	ORR	CRR	N	ORR	CRR	N	ORR	CRR
Total	21	12(57.1%)	4(19%)	10	6(60%)	2(20%)	11	6(54.5%)	2(18.2%)
CsA	6	3(50%)	0	3	2(66.7%)	0	3	1(33.3%)	0
CsA+Steroids	10	6(60%)	1(10%)	5	3(60%)	1(20%)	5	3(60%)	0
CsA+CTX+Steroids	5	3(60%)	3(60%)	2	1(50%)	1 (50%)	3	2(66.7%)	2(66.7%)

### 3.5 Comparison of clinical data between T-LGLL-PRCA and T-LGLL-non-PRCA

To further investigate factors associated with the development of T-LGLL-PRCA, we conducted a comparative analysis of relevant clinical parameters between the two groups. The results showed that circulating T-LGL (*P* = 0.040), HB (*P* < 0.001), HCT (*P* = 0.003), RNC (*P* < 0.001), IRF (*P* = 0.002), EPO levels>750 mIU/mL (*P* = 0.009) and TCR subtype (*P* = 0.049) differed significantly between the two groups (*P* < 0.05) (**Tables 9**–**11**).

**Table 9:** Comparison of baseline data between the PRCA and Non-PRCA groups.

Items	PRCA(n=15)	Non-PRCA(n=25)	*P* value
Age	68 (64, 73)	61 (55, 69)	.085
Sex (n,%)			.332
Male	5/15 (33.3%)	13/25 (52%)	
Female	10/15 (66.7%)	12/25 (48%)	
Circulating T-LGL	0.79 ± 0.81	1.62 ± 2.60	.040
BM-T-LGL	19.96±20.55	20.20±18.22	.368
WBC	3.81 (3.09, 4.42)	4.52 (2.11, 7.95)	.539
ANC	1.39 (1.10, 2.62)	1.23 (0.53, 2.36)	.468
ALC	1.9 (0.91, 2.93)	1.41 (0.88, 5.10)	.539
PLT	250 (119, 323)	239 (176, 296)	.911
HB	42 (23, 63)	88 (59, 107)	<.001
HCT	17.9 (10.1, 20.4)	25.8 (18.35, 31.95)	.003
RNC	6.10 ± 3.31	49.64 ± 47.22	<.001
IRF	6,5 (0.9, 14)	18.5 (7.65, 26.2)	.002
EPO (mIU/mL)			.009
>750	11/15 (73.3%)	7/25 (28%)	
≤750	4/15 (26.7%)	18/25 (72%)	
Splenomegaly (n,%)			.381
Yes	1/15 (6.7%)	5/25 (20%)	
No	14/15 (93.3%)	20/25 (80%)	
CD3+/CD8+			1.000
Yes	13/15 (86.7%)	22/25 (88%)	
No	2/15 (13.3%)	3/25 (12%)	

**Table 10:** Comparison of immune factors between the PRCA and Non-PRCA groups.

Items	PRCA(n=8)	Non-PRCA(n=10)	*P* value
RF (n,%)			1.000
Pos.	1/8 (12.5%)	2/10 (20%)	
Neg.	7/8 (87.5%)	8/10 (80%)	
ANA (n,%)			.314
Pos.	7/8 (87.5%)	6/10 (60%)	
Neg.	1/8 (12.5%)	4/10 (40%)	
EBV (n,%)			.152
Pos.	7/8 (87.5%)	5/10 (50%)	
Neg.	1/8 (12.5%)	5/10 (50%)	

**Table 11:** Comparison of TCR Subtype between the PRCA and Non-PRCA groups.

Items	PRCA(n=11)	Non-PRCA(n=14)	*P* value
TCR Subtype			.049
TCR-αβ (+)	4/11 (36.4%)	11/14 (78.6%)	
TCR-γδ (+)	7/11 (63.6%)	3/14 (21.4%)	

### 3.6 Independent influencing factors of T-LGLL-PRCA

​Binary logistic regression analysis was performed to systematically identify independent factors associated with the development of T-LGLL-PRCA. Univariate logistic regression analysis showed that HB (OR:0.926;*P* = 0.003), HCT(OR:0.837;*P* = 0.006), EPO>750mIU/mL (OR:7.071;*P* = 0.008), RNC (OR:0.682;*P* = 0.027), and IRF (OR:0.857;*P* = 0.007) were significantly associated with the development of T-LGLL-PRCA. Multivariate logistic regression analysis further revealed that RNC (OR:0.590;*P* = 0.048) was the only independent factor associated with the development of T-LGLL-PRCA (*P* < 0.05) (**Table 12**).

**Table 12:** Univariable and multivariable logistic regression analysis of factors affecting T-LGLL-PRCA.

Variables	Univariable analysis		Multivariable analysis	
	HR (95% CI)	*P* value	HR (95% CI)	*P* value
Age≥65 (Yes *vs.* No)	2.979(0.744-11.931)	.123		
Male (Female *vs.* male)	2.167(0.573-8.190)	.254		
Circulating T-LGL	0.721(0.410-1.270)	.258		
BM-T-LGL	0.999(0.966-1.034)	.969		
WBC	0.862(0.690-1.076)	.189		
ANC	0.917(0.642-1.310)	.635		
ALC	0.776(0.539-1.116)	.171		
PLT	0.999(0.993-1.006)	.833		
HB	0.926(0.880-0.974)	.003	0.915(0.797-1.051)	.209
HCT	0.837(0.737-0.950)	.006	0.845(0.516-1.385)	.505
EPO>750 (Yes *vs.* No)	7.071(1.676-29.828)	.008	0.294(0.018-4.788)	.390
RNC	0.682(0.485-0.957)	.027	0.590(0.350-0.996)	.048
IRF	0.857(0.766-0.958)	.007	1.323(0.909-1.927)	.144
Splenomegaly (Yes *vs.* No)	0.286(0.030-2.719)	.276		
CD3+/CD8+ (Yes *vs.* No)	0.886(0.130-6.022)	.902		

## 4 Discussion

Historically, thymoma was considered the predominant underlying cause of PRCA. However, recent clinical observations in Asian populations indicate that T-LGLL has surpassed thymoma as the most common underlying condition associated with PRCA, although relevant reports remain limited and are largely restricted to isolated case descriptions or secondary findings. Furthermore, most available studies originate from Western populations, and differences in region, ethnicity, and diagnostic criteria hinder the ability to obtain reliable assessments of clinical manifestations and therapeutic recommendations. Against this background, the present study adopted a cohort-based design focusing specifically on T-LGLL-PRCA rather than relying on scattered case reports. We retrospectively included 40 patients with T-LGLL from a single center and stratified them into T-LGLL-PRCA and T-LGLL–Non-PRCA groups according to PRCA status at diagnosis. By analyzing and comparing the two groups, we systematically characterized the key clinical features, laboratory parameters, and prognosis-related factors of this rare disease subtype. This approach minimizes the selection bias and informational limitations inherent to case reports, yielding conclusions with greater statistical robustness and clinical relevance, thereby providing more reliable evidence-based support for the management of T-LGLL-PRCA. Nevertheless, given the rarity of T-LGLL, these findings are derived from a limited sample within a single center, and larger multicenter studies are needed to further validate and refine these observations.

Although numerous authoritative experts and researchers in the LGL field have proposed widely cited diagnostic criteria for T-LGLL, it must be acknowledged that no fully standardized diagnostic system or universally accepted expert consensus currently exists at the international level. At present, the diagnosis and classification of LGLL rely primarily on clinical manifestations, immunophenotyping of peripheral blood or bone marrow cells, and assessment of T-cell receptor clonality. Historically, diagnosis required an elevated proportion of peripheral LGLs with an absolute count >2 × 10^9^/L persisting for more than six months; however, contemporary diagnostic approaches place greater emphasis on demonstrating clonal proliferation of T-LGLs with characteristic immunophenotypes (4). For example, Lamy et al. reported that 25–30% of T-LGLL cases do not exhibit a marked increase in peripheral LGLs and often present with counts <0.5 × 10^9^/L (1). Semenzato et al. further showed that patients with LGL counts <2.0 × 10^9^/L display clinical and laboratory features similar to those with higher counts, and that monoclonal TCR rearrangements, bone marrow immunophenotyping, and disease evolution over six months also support the diagnosis of LGLL (29). Incorporating these findings, together with the 2022 fifth edition of the WHO Classification and the 2024 Blood review “A Modern View of LGL Leukemia, “we revised the diagnostic criteria by setting the threshold for peripheral T-LGLs at ≥0.5 × 10^9^/L. When counts fall below this threshold, diagnosis may rely on cytologic immunophenotyping and/or monoclonal TCR detection, supplemented by bone marrow biopsy with immunohistochemistry (15). Furthermore, T-LGLL must be distinguished from reactive T-LGL proliferations, which are typically polyclonal, transient, and associated with infectious or autoimmune conditions. In cases of diagnostic uncertainty, re-evaluation after six months—including clinical assessment, hematologic parameters, and clonality testing—may help differentiate T-LGLL from reactive proliferations.

The pathogenesis of T-LGLL–associated PRCA is thought to involve a bone marrow failure syndrome primarily mediated by CD3^+^CD8^+^ T-LGLL cells, which suppress erythroid progenitor function (9, 30). Based on this mechanism, we initially hypothesized that a higher burden of T-LGLL cells would correlate with an increased risk of secondary PRCA. However, our findings demonstrated no significant association between elevated proportions of T-LGLL cells in either peripheral blood or bone marrow and the occurrence of PRCA, a finding that appears inconsistent with the established pathogenic model. This discrepancy may reflect the relatively small sample size of our cohort, but it is also possible that the pathophysiological relationship between clonal T-LGLL expansion and erythroid suppression is more complex than previously understood, underscoring the need for validation in larger, multicenter studies. Furthermore, the clinical presentation of T-LGLL-PRCA closely resembles that of general PRCA populations, including common anemia-related manifestations such as dizziness and fatigue, as well as frequent EBV infection and ANA positivity. These observations raise the possibility that a substantial proportion of cases currently classified as primary or idiopathic PRCA may, in fact, represent unrecognized T-LGLL–associated disease. Given the diagnostic limitations inherent to these conditions, the true incidence of T-LGLL-PRCA is likely underestimated in current clinical studies. In addition, we observed low rates of coexisting rheumatic immune disorders and splenomegaly among patients with T-LGLL-PRCA, findings that are consistent with domestic research cohorts but differ from Western reports (8, 31). These discrepancies may reflect geographic and ethnic variation or differences in diagnostic practices across clinical centers, potentially contributing to selection bias.

The principal pathological feature of T-LGLL–associated PRCA is the selective failure of erythropoiesis within the bone marrow. Erythroid progenitor proliferation and differentiation are impaired, leading to failed maturation into downstream erythroid lineages and ultimately resulting in insufficient production of mature erythrocytes in the peripheral circulation, whereas granulopoiesis and megakaryopoiesis remain largely preserved (1, 4). In this study, clinical indicators reflecting distinct stages of erythroid differentiation were integrated with the underlying pathogenesis and developmental trajectory of erythropoiesis in T-LGLL–PRCA, with the aim of systematically delineating its clinicopathological features and establishing a comprehensive evaluative framework. Our findings further refined the stages at which erythroid maturation is disrupted, demonstrating markedly reduced HB and HCT levels in the T-LGLL–PRCA group, indicative of impaired terminal erythrocyte production. The pronounced reductions in RNC and IRF suggested that erythroid maturation is arrested prior to the stage of immature reticulocyte formation. Importantly, RNC is directly linked to key pathological events in PRCA and serves not only as a diagnostic marker but also as an indicator of disease activity and therapeutic response, with strong associations with disease onset, progression, and prognosis. Consistent with our identification of RNC as an independent influencing factor, it may serve as a central indicator for assessing therapeutic response in T-LGLL–PRCA. When treatment is effective and erythropoiesis begins to recover, RNC typically rises earlier than HB, signaling the release of erythroid differentiation blockade. Furthermore, the pathological process of T-LGLL–PRCA may be elucidated more precisely by evaluating defects in erythroid progenitor proliferation and differentiation. Future studies incorporating direct quantification of BFU-E and CFU-E in the bone marrow of T-LGLL patients may help define the precise locus of hematopoietic blockade, thereby improving diagnostic accuracy and informing clinical management.

Previous studies on the pathogenesis of T-LGLL have suggested that expansion of clonal hematopoiesis in the bone marrow may either trigger clonal cytotoxicity or serve as a target for dysregulated T-cell responses. Genetic mutations may contribute significantly to this process, particularly in cases complicated by PRCA (32, 33). In our cohort, TET2 and DNMT3A mutations were relatively common among T-LGLL–PRCA patients who underwent mutation testing, consistent with findings by Fujishima et al., who reported that 4 of 11 patients with acquired PRCA harbored TET2 or DNMT3A mutations, suggesting a potential association between these mutations and impaired erythropoiesis (34). Moreover, recent studies indicate that TET2 mutations occur in up to 23% of LGLL patients, making them the most frequent genetic alteration in this disease and linking them to expanded malignant clonal hematopoiesis and neutropenia (33, 35). These observations suggest that TET2 mutations may promote proliferation of clonal hematopoietic cells and indirectly enhance aberrant T-cell activation and cytotoxicity. However, the direct contribution of TET2 mutations to erythroid failure in T-LGLL–PRCA remains unclear. It also remains uncertain whether TET2 or DNMT3A mutations function as true driver events or merely represent co-evolving byproducts of long-standing clonal hematopoiesis. Future studies integrating single-cell sequencing, epigenetic landscape profiling, and functional assays will be essential for elucidating the mechanistic roles of TET2 and DNMT3A in erythroid failure, representing a key direction for future research.

In studies of the pathogenesis of T-LGLL–PRCA, dysregulated immune modulation has consistently remained a central focus of investigation. Recent evidence suggests that in T-LGLL arising in the context of autoimmune disorders, aberrant inflammatory cytokine secretion by activated T cells may represent a key mechanism driving erythroid failure, involving cytokines such as IL-2, IL-6, TNF-α, and IFN-γ (36, 37). From a clinical perspective, cytokine profiling offers several potential advantages in the management of T-LGLL–PRCA, including diagnostic and differential diagnostic value, monitoring of therapeutic response, and prognostic assessment. Cytokine profiling of 11 patients with T-LGLL–PRCA at our institution further supports this mechanism, as IL-2 and TNF-α levels were significantly elevated in six patients, whereas no notable abnormalities were detected in IL-1β, IL-6, IL-8, or IL-10. Nevertheless, this study is limited by its small sample size and the absence of correlation analyses linking cytokine profiles with clinical phenotypes. Future research should expand cohort size and incorporate flow cytometric assessment of cytokine secretion profiles in clonal T cells to elucidate the mechanistic roles and clinical utility of inflammatory cytokines in T-LGLL–PRCA.

T-LGLL is a clinically and biologically heterogeneous disorder that encompasses a broad spectrum of manifestations. When complicated by the rare entity of secondary PRCA, conducting randomized clinical trials becomes particularly challenging; consequently, the management of refractory cases relies heavily on empirical experience, case reports, and case series. Several potentially useful therapeutic agents—such as anti-thymocyte globulin, alemtuzumab, the IL-15 inhibitor BNZ-1, linperlisib, thalidomide, sirolimus, fludarabine, and TCR Vβ monoclonal antibodies—have been evaluated in clinical trials for patients with relapsed or refractory T-LGLL after first-line therapy (38–41). For T-LGLL–PRCA specifically, ruxolitinib has been reported as a potentially effective agent, likely through inhibition of aberrant JAK/STAT signaling and mitigation of erythroid suppression, thereby providing a novel rationale for targeted intervention (42, 43). Overall, as understanding of the immunopathogenesis of T-LGLL continues to advance and additional targeted therapies and immunomodulatory strategies emerge, the development of individualized, mechanism-based treatment approaches for T-LGLL and its associated PRCA holds considerable promise. These innovative therapeutic modalities merit further evaluation and may ultimately improve long-term outcomes for affected patients.

## Data Availability

The raw data supporting the conclusions of this article will be made available by the authors, without undue reservation.

## References

[1] Lamy T Moignet A Loughran TP Jr . LGL leukemia: from pathogenesis to treatment. Blood. (2017) 129:1082–94. doi: 10.1182/blood-2016-08-692590, PMID: 28115367

[2] Matutes E . The 2017 WHO update on mature T- and natural killer (NK) cell neoplasms. Int J Lab Hematol. (2018) 40 Suppl 1:97–103. doi: 10.1111/ijlh.12817, PMID: 29741263

[3] Durrani J Awada H Kishtagari A Visconte V Kerr C Adema V . Large granular lymphocytic leukemia coexists with myeloid clones and myelodysplastic syndrome. Leukemia. (2020) 34:957–62. doi: 10.1038/s41375-019-0601-y, PMID: 31624375 PMC8370475

[4] Marchand T Lamy T Loughran TP Jr . A modern view of LGL leukemia. Blood. (2024) 144:1910–23. doi: 10.1182/blood.2023021790, PMID: 38848524

[5] Barila G Calabretto G Teramo A Vicenzetto C Gasparini VR Semenzato G . T cell large granular lymphocyte leukemia and chronic NK lymphocytosis. Best Pract Res Clin Haematol. (2019) 32:207–16. doi: 10.1016/j.beha.2019.06.006, PMID: 31585621

[6] Cheon H Dziewulska KH Moosic KB Olson KC Gru AA Feith DJ . Advances in the diagnosis and treatment of large granular lymphocytic leukemia. Curr Hematol Malig Rep. (2020) 15:103–12. doi: 10.1007/s11899-020-00565-6, PMID: 32062772 PMC7234906

[7] Magnano L Rivero A Matutes E . Large granular lymphocytic leukemia: current state of diagnosis, pathogenesis and treatment. Curr Oncol Rep. (2022) 24:633–44. doi: 10.1007/s11912-021-01159-y, PMID: 35212923

[8] Dong N Castillo Tokumori F Isenalumhe L Zhang Y Tandon A Knepper TC . Large granular lymphocytic leukemia -A retrospective study of 319 cases. Am J Hematol. (2021) 96:772–80. doi: 10.1002/ajh.26183, PMID: 33819354

[9] Liu M He X Zhang H Liu Y Yang L Wei Y . Aberrant hematopoiesis and CD8(+) T-cell activation in thymoma-associated pure red cell aplasia. Thorac Cancer. (2025) 16:e70046. doi: 10.1111/1759-7714.70046, PMID: 40109027 PMC11923450

[10] Means Robert T . Pure red cell aplasia: The second hundred years. Am J Of Med Sci. (2023) 366:160–6. doi: 10.1016/j.amjms.2023.06.009, PMID: 37327996

[11] Salama Y Zhao F Oliveira JL Yuan J Jevremovic D Go RS . Isolated anemia in patients with large granular lymphocytic leukemia (LGLL). Blood Cancer J. (2022) 12:30. doi: 10.1038/s41408-022-00632-6, PMID: 35194022 PMC8863822

[12] Ogbue O Kewan T Bravo-Perez C Unlu S Kawashima N Williams ND . Hemolytic versus malproductive anemia in large granular lymphocytic leukemia. Leukemia. (2024) 38:1839–42. doi: 10.1038/s41375-024-02323-6, PMID: 38982262 PMC11286512

[13] Gurnari C Maciejewski JP . How I manage acquired pure red cell aplasia in adults. Blood. (2021) 137:2001–9. doi: 10.1182/blood.2021010898, PMID: 33657207 PMC8057257

[14] Vicenzetto C Gasparini VR Barila G Teramo A Calabretto G Rampazzo E . Pro-inflammatory cells sustain leukemic clonal expansion in T-cell large granular lymphocyte leukemia. Haematologica. (2024) 109:163–74. doi: 10.3324/haematol.2022.282306, PMID: 37439335 PMC10772499

[15] Baram DV Asaulenko ZP Spiridonov IN Krivolapov YA . WHO classification of tumors of hematopoietic and lymphoid tissues, 2022 (5th edition): lymphoid tumors. Arkh Patol. (2023) 85:24–31. doi: 10.17116/patol20238504124, PMID: 37530187

[16] Red Blood Cell Disease Group, C. S. o. H. C. M. A . Chinese expert consensus on the diagnosis and treatment of acquired pure red cell aplasia (2020). Zhonghua Xue Ye Xue Za Zhi. (2020) 41:177–84. doi: 10.3760/cma.j.issn.0253-2727.2020.03.001, PMID: 32311886 PMC7357928

[17] Brugnara C . Reticulocyte cellular indices: a new approach in the diagnosis of anemias and monitoring of erythropoietic function. Crit Rev In Clin Lab Sci. (2000) 37:93–130. doi: 10.1080/10408360091174196, PMID: 10811141

[18] Wollmann M Gerzson BMC Schwert V Figuera RW de Oliveira Ritzel G . Reticulocyte maturity indices in iron deficiency anemia. Rev Bras Hematologia e Hemoterapia. (2014) 36:25–8. doi: 10.5581/1516-8484.20140009, PMID: 24624032 PMC3948662

[19] Soliman D Sallam S Akiki S Mudawi D Ibrahim F . T-cell large granular lymphocytic leukemia with extremely rare immunophenotype (CD4/CD8 double-positive) followed by multiple myeloma diagnosis. Case Rep Hematol. (2020) 2020:8839144. doi: 10.1155/2020/8839144, PMID: 32855829 PMC7443251

[20] Chen Y-H Chadburn A Evens AM Winter JN Gordon LI Chenn A . Clinical, morphologic, immunophenotypic, and molecular cytogenetic assessment of CD4-/CD8-γδ T-cell large granular lymphocytic leukemia. Am J Of Clin Pathol. (2011) 136:289–99. doi: 10.1309/AJCPTFFQ18JMYKDF, PMID: 21757603

[21] Giudice V D’Addona M Montuori N Selleri C . The value of flow cytometry clonality in large granular lymphocyte leukemia. Cancers. (2021) 13(18). doi: 10.3390/cancers13184513, PMID: 34572739 PMC8468916

[22] El-Sharkawi D Dearden C . Diagnosis and management of large granular lymphocytic leukaemia and its differentiation from T cell clones of uncertain significance. Hematol Oncol. (2025) 43 Suppl 2:e70076. doi: 10.1002/hon.70076, PMID: 40517544

[23] Semenzato G Calabretto G Teramo A Gasparini VR Rampazzo E Barila G . The constitutive activation of STAT3 gene and its mutations are at the crossroad between LGL leukemia and autoimmune disorders. Blood Cancer J. (2024) 14:13. doi: 10.1038/s41408-024-00977-0, PMID: 38238319 PMC10796758

[24] O'Keefe CL Plasilova M Wlodarski M Risitano AM Rodriguez AR Howe E . Molecular analysis of TCR clonotypes in LGL: a clonal model for polyclonal responses. J Immunol. (2004) 172:1960–9. doi: 10.4049/jimmunol.172.3.1960, PMID: 14734782

[25] Kawashima N Gurnari C Bravo-Perez C Kubota Y Pagliuca S Guarnera L . Clonal hematopoiesis in large granular lymphocytic leukemia. Leukemia. (2025) 39:451–9. doi: 10.1038/s41375-024-02460-y, PMID: 39572711

[26] Sanikommu SR Clemente MJ Chomczynski P Afable MG 2nd Jerez A Thota S . Clinical features and treatment outcomes in large granular lymphocytic leukemia (LGLL). Leuk Lymphoma. (2018) 59:416–22. doi: 10.1080/10428194.2017.1339880, PMID: 28633612 PMC8694069

[27] Sabnani I Tsang P . Are clonal T-cell large granular lymphocytes to blame for unexplained haematological abnormalities? Br J Haematol. (2007) 136:30–7. doi: 10.1111/j.1365-2141.2006.06374.x, PMID: 17092307

[28] Ullah F Markouli M Orland M Ogbue O Dima D Omar N . Large granular lymphocytic leukemia: clinical features, molecular pathogenesis, diagnosis and treatment. Cancers (Basel). (2024) 16(7). doi: 10.3390/cancers16071307, PMID: 38610985 PMC11011145

[29] Steinway SN LeBlanc F Loughran TP Jr . The pathogenesis and treatment of large granular lymphocyte leukemia. Blood Rev. (2014) 28:87–94. doi: 10.1016/j.blre.2014.02.001, PMID: 24679833 PMC4155502

[30] Hirokawa M Sawada K Fujishima N Teramura M Bessho M Dan K . Long-term outcome of patients with acquired chronic pure red cell aplasia (PRCA) following immunosuppressive therapy: a final report of the nationwide cohort study in 2004/2006 by the Japan PRCA collaborative study group. Br J Haematol. (2015) 169:879–86. doi: 10.1111/bjh.13376, PMID: 25807974

[31] Dinmohamed AG Brink M Visser O Jongen-Lavrencic M . Population-based analyses among 184 patients diagnosed with large granular lymphocyte leukemia in the Netherlands between 2001 and 2013. Leukemia. (2016) 30:1449–51. doi: 10.1038/leu.2016.68, PMID: 27055870

[32] Marchand T Pastoret C Moignet A Roussel M Lamy T . Large granular lymphocyte leukemia: a clonal disorder with autoimmune manifestations. Hematology-American Soc Hematol Educ Program. (2024) 2024:143–9. doi: 10.1182/hematology.2024000539, PMID: 39644019 PMC11665628

[33] Kawakami T Kawakami F Matsuzawa S Yamane T Mizuno Y Asakura A . Mutational heterogeneities in STAT3 and clonal hematopoiesis-related genes in acquired pure red cell aplasia. Ann Hematol. (2025) 104:1471–9. doi: 10.1007/s00277-025-06356-4, PMID: 40202536 PMC12031804

[34] Fujishima N Kohmaru J Koyota S Kuba K Saga T Omokawa A . Clonal hematopoiesis in adult pure red cell aplasia. Sci Rep. (2021) 11:2253. doi: 10.1038/s41598-021-81890-5, PMID: 33500526 PMC7838416

[35] Olson TL Cheon H Xing JC Olson KC Paila U Hamele CE . Frequent somatic TET2 mutations in chronic NK-LGL leukemia with distinct patterns of cytopenias. Blood. (2021) 138:662–73. doi: 10.1182/blood.2020005831, PMID: 33786584 PMC8394905

[36] Shvidel L Duksin C Tzimanis A Shtalrid M Klepfish A Sigler E . Cytokine release by activated T-cells in large granular lymphocytic leukemia associated with autoimmune disorders. Hematol J. (2002) 3:32–7. doi: 10.1038/sj.thj.6200149, PMID: 11960393

[37] Isabelle C Boles A Chakravarti N Porcu P Brammer J Mishra A . Cytokines in the pathogenesis of large granular lymphocytic leukemia. Front Oncol. (2022) 12:849917. doi: 10.3389/fonc.2022.849917, PMID: 35359386 PMC8960188

[38] Zhang L Qiu C Li R Shen Y Tian L Chang H . KLRG1 re-defines a leukemic clone of CD8 effector T cells sensitive to PI3K inhibitor in T cell large granular lymphocytic leukemia. Cell Rep Med. (2025) 6(4). doi: 10.1016/j.xcrm.2025.102036, PMID: 40147444 PMC12047471

[39] Yu Y Li Y Cui R Yan Y Li F Chen Y . Thalidomide-based regimen shows promising efficacy in large granular lymphocytic leukemia: a multicenter phase II study. Signal Transduct Target Ther. (2025) 10:85. doi: 10.1038/s41392-025-02164-4, PMID: 40069155 PMC11897152

[40] Brammer JE Ballen K Sokol L Querfeld C Nakamura R Mishra A . Effective treatment with the selective cytokine inhibitor BNZ-1 reveals the cytokine dependency of T-LGL leukemia. Blood. (2023) 142:1271–80. doi: 10.1182/blood.2022017643, PMID: 37352612 PMC10613725

[41] Prince HM . Blocked addiction to IL-15 for treating T-LGLL. Blood. (2023) 142:1258–60. doi: 10.1182/blood.2023021476, PMID: 37824162

[42] Gong Y Li Y Chen X Yang H Zhang Y He G . Refractory pure red cell aplasia associated with T-cell large granular lymphocyte leukemia treated by ruxolitinib. Ann Hematol. (2024) 103:3239–42. doi: 10.1007/s00277-024-05856-z, PMID: 38935319

[43] Marchand T Pastoret C Damaj G Lebouvier A Herbaux C Moignet A . Efficacy of ruxolitinib in the treatment of relapsed/refractory large granular lymphocytic leukaemia. Br J Haematol. (2024) 205:915–23. doi: 10.1111/bjh.19476, PMID: 38639192

